# Kinship practices in Early Iron Age southeast Europe: genetic and isotopic analysis of burials from the Dolge njive barrow cemetery, Dolenjska, Slovenia

**DOI:** 10.15184/aqy.2023.2

**Published:** 2023-02-17

**Authors:** Ian Armit, Claire-Elise Fischer, Hannah Koon, Rebecca Nicholls, Iñigo Olalde, Nadin Rohland, Jo Buckberry, Janet Montgomery, Philip Mason, Matija Črešnar, Lindsey Büster, David Reich

**Affiliations:** 1 Department of Archaeology, University of York, King’s Manor, York, United Kingdom YO1 7EP; 2 School of Archaeological and Forensic Sciences, University of Bradford, Bradford, West Yorkshire, United Kingdom BD7 1DP; 3 BIOMICs Research Group, University of the Basque Country UPV/EHU, 01006 Vitoria-Gasteiz, Spain.; 4 Ikerbasque – Basque Foundation of Science, 48009 Bilbao, Spain.; 5 Department of Genetics, Harvard Medical School, 77 Avenue Louis Pasteur, Boston, MA 02115, United States; 6 Department of Archaeology, Durham University, South Road, Durham, United Kingdom DH1 3LE; 7 Institute for the Protection of Cultural Heritage of Slovenia, Poljanska ulica 40, 1000 Ljubljana, Slovenia; 8 Department of Archaeology, Faculty of Arts, University of Ljubljana, Aškerčeva 2, 1000 Ljubljana, Slovenia; 9 School of Humanities and Educational Studies, Canterbury Christchurch University, North Holmes Road, Canterbury, Kent CT1 1QU; 10 Department of Human Evolutionary Biology, Harvard University, Cambridge, MA 02138, United States; 11 The Howard Hughes Medical Institute, Harvard Medical School, 77 Avenue Louis Pasteur, Boston, MA 02115, United States; 12 Broad Institute of MIT and Harvard, Cambridge, MA 02142, United States

**Keywords:** ancient DNA, Iron Age, Slovenia, barrows, isotope analysis, kinship

## Abstract

DNA analysis demonstrates that all seven individuals buried in an Early Iron Age barrow at Dolge njive, southeast Slovenia, are close biological relatives. Although group composition does not suggest strict adherence to a patrilineal or matrilineal kinship system, the funerary tradition appears highly gendered, with family links through both the male and female line being important in structuring communities. We explore the implications for our understandings of kinship and funerary practices in Early Iron Age southeast Europe.

## Introduction

The beginning of the Early Iron Age (c. 800–450 BC) in southeast Europe was accompanied by significant social changes, many of them apparently related to a growing intensity of contacts and exchange between communities around the head of the Adriatic, and with the urbanising societies of the wider Mediterranean world. These changes are marked in eastern Slovenia, as well as in the broader area between the Eastern Alps and Western Pannonia, by the emergence of new centres of population comprising large hillforts associated with extensive barrow cemeteries and, in some cases, evidence for iron-working (e.g. Teržan 1990; Mason 1996; Dular & Tecco Hvala 2007; Mason & Mlekuž 2016; Črešnar & Mele 2019; Črešnar et al. 2020). In the Early Iron Age Dolenjska group (southeast Slovenia and northern Croatia), which is the focus of this paper, funerary rites shifted from cremation burials in flat cemeteries to inhumation, usually comprising multiple graves under a substantial earthen barrow, often with significant quantities of grave goods. These new centres can be linked to the emergence of extended hierarchies that developed to control and exploit production and inter-regional trade in, for example, iron, salt and amber.

Although it has been suggested that burial in these barrows might have been based on familial links, with individual barrows being associated with specific lineages (Dular & Tecco Hvala 2007: 123–6, 237–45; Teržan 2010), this has been hard to demonstrate using traditional archaeological techniques. As part of the HERA-funded ENTRANS (Encounters and Transformations in Iron Age Europe) Project (Armit et al. 2014; 2016), osteological and isotope analysis was applied to sites in the region, with further aDNA analysis obtained through the COMMIOS (Communities and Connectivities: Iron Age Britons and their Continental Neighbours) Project. This paper details the results of work on one of these sites, the Dolge njive barrow cemetery, and examines their implications for our wider understanding of human mobility and family structure during this dynamic period of southeast European prehistory.

## The Dolge njive barrow cemetery

**Figure 1.**
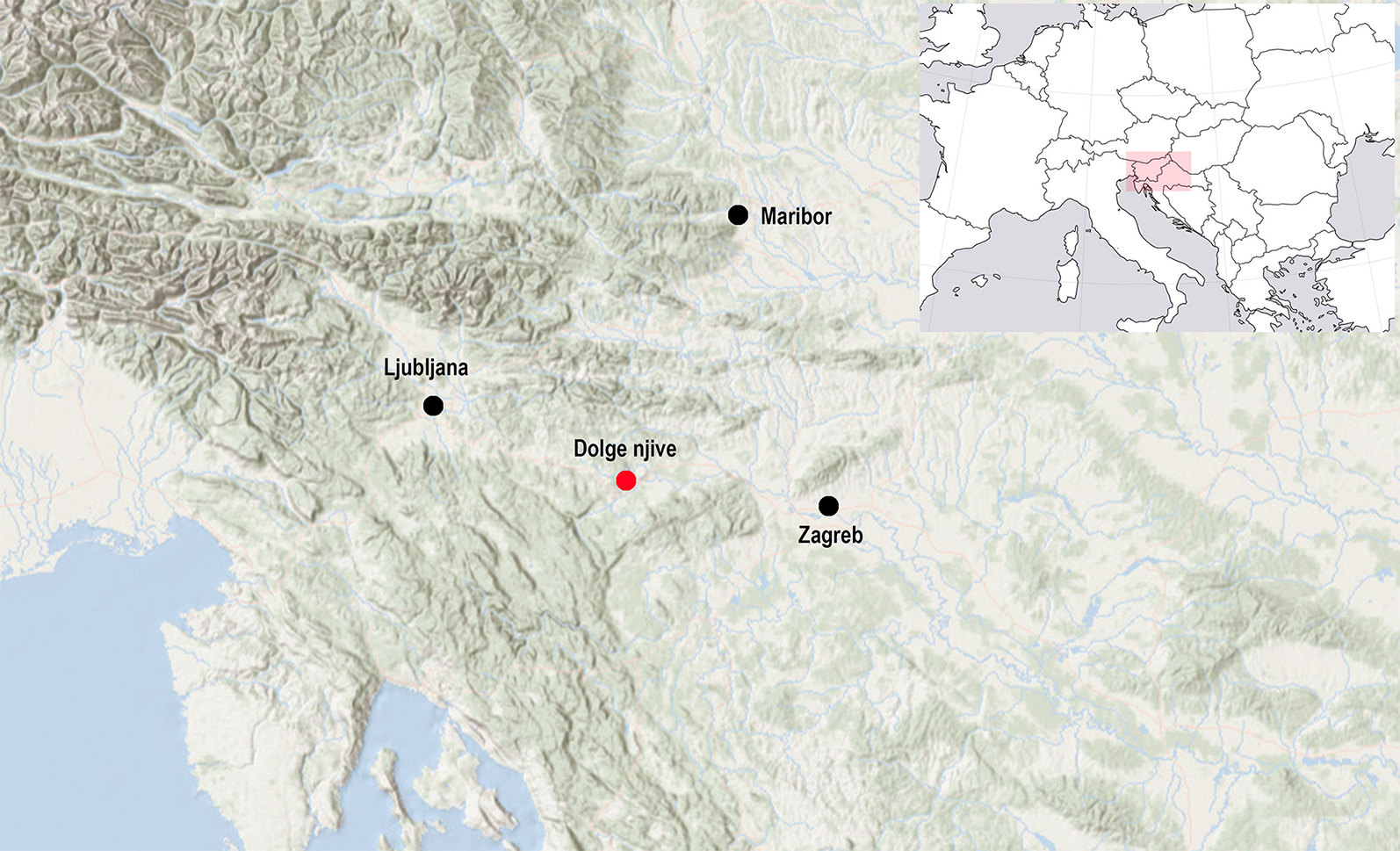
Location map

The Dolge njive cemetery forms part of one of the largest mortuary complexes of the Early Iron Age Dolenjska group, which stretches over southeast Slovenia and part of northern Croatia (Figure 1). The complex centres on the large (12.68 ha) hillfort at Veliki Vinji vrh and comprises an estimated 145 barrows. Four main groups ascend to the northwestern entrance of the hillfort from the Topličica valley, while a further 45 dispersed barrows, erected individually or in smaller groups, extend across a wider area of over 25 km^2^. Many of these barrows were excavated in the late nineteenth century with a relatively poor standard of field-recording and documentation; modern excavation has confirmed, however, that skeletal remains in the area are generally very poorly preserved or absent (Dular & Tecco Hvala 2007: 191; Mason & Mlekuž 2016).

**Figure 2.**
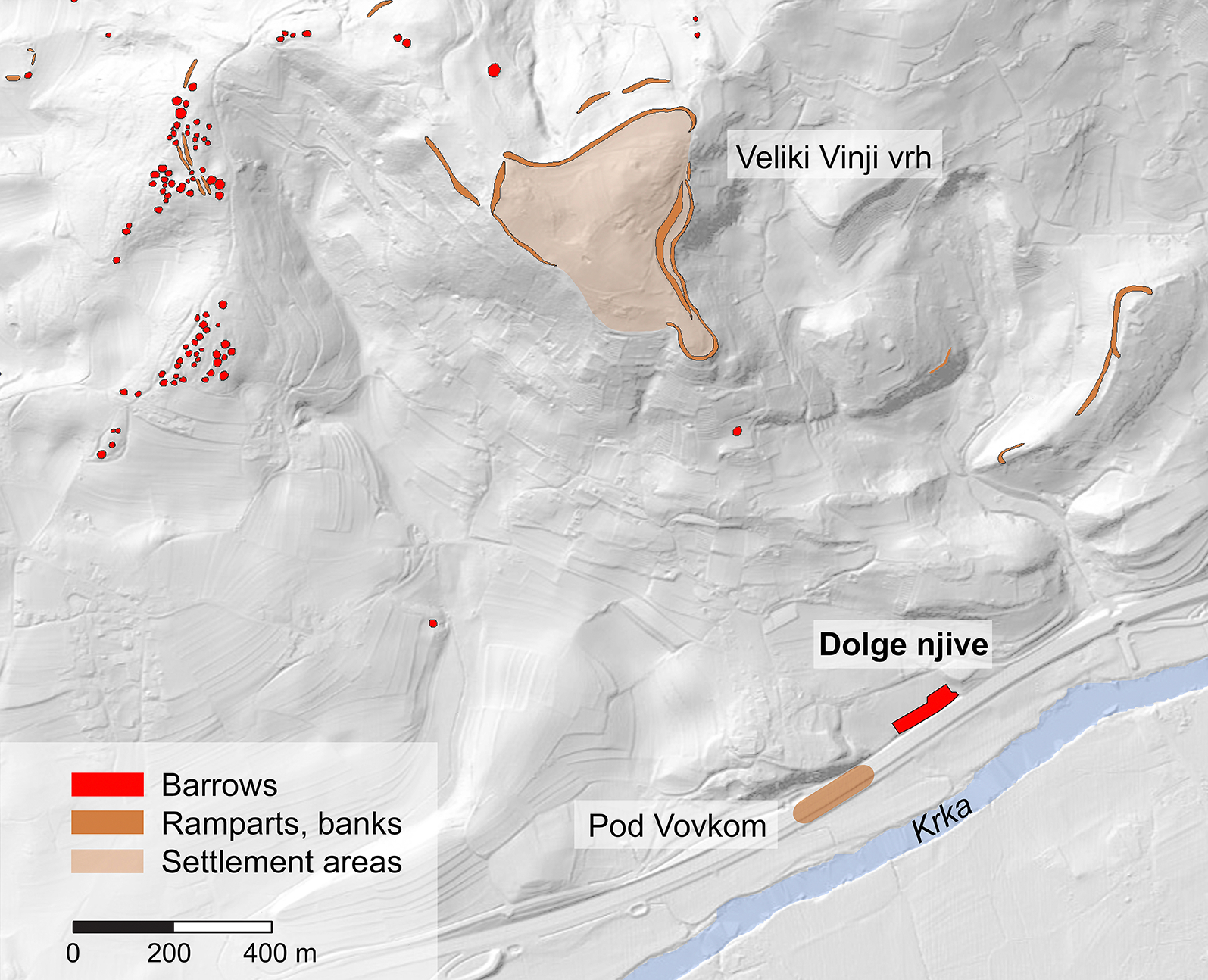
Plan of the Vinji vrh complex, with the Dolge njive cemetery indicated.

The Dolge njive cemetery itself is located between two deeply incised valleys at the foot of the Vinji vrh massif, southeast of the hillfort (Figure 2). Excavations in 2002, in advance of motorway construction, revealed the poorly preserved remains of three Early Iron Age barrows. Two of these were constructed on the site of Late Bronze Age cremation platforms, whilst the third was located a short distance to the east and connected to the others by a Late Bronze Age hollow-way with associated deposits of cremated bone (Mason 2005; Mason & Mlekuž 2016). The remains of an Early Iron Age farmstead or small settlement, comprising two cobbled surfaces and two apparently domestic structures, were discovered at Pod Vovkom to the southwest of the site (Križ 2005; Figure 2). Its location was undoubtedly influenced by the proximity of the Krka River, but may also have taken advantage of one of the possible route-ways from the valley to the hillfort.

**Figure 3.**
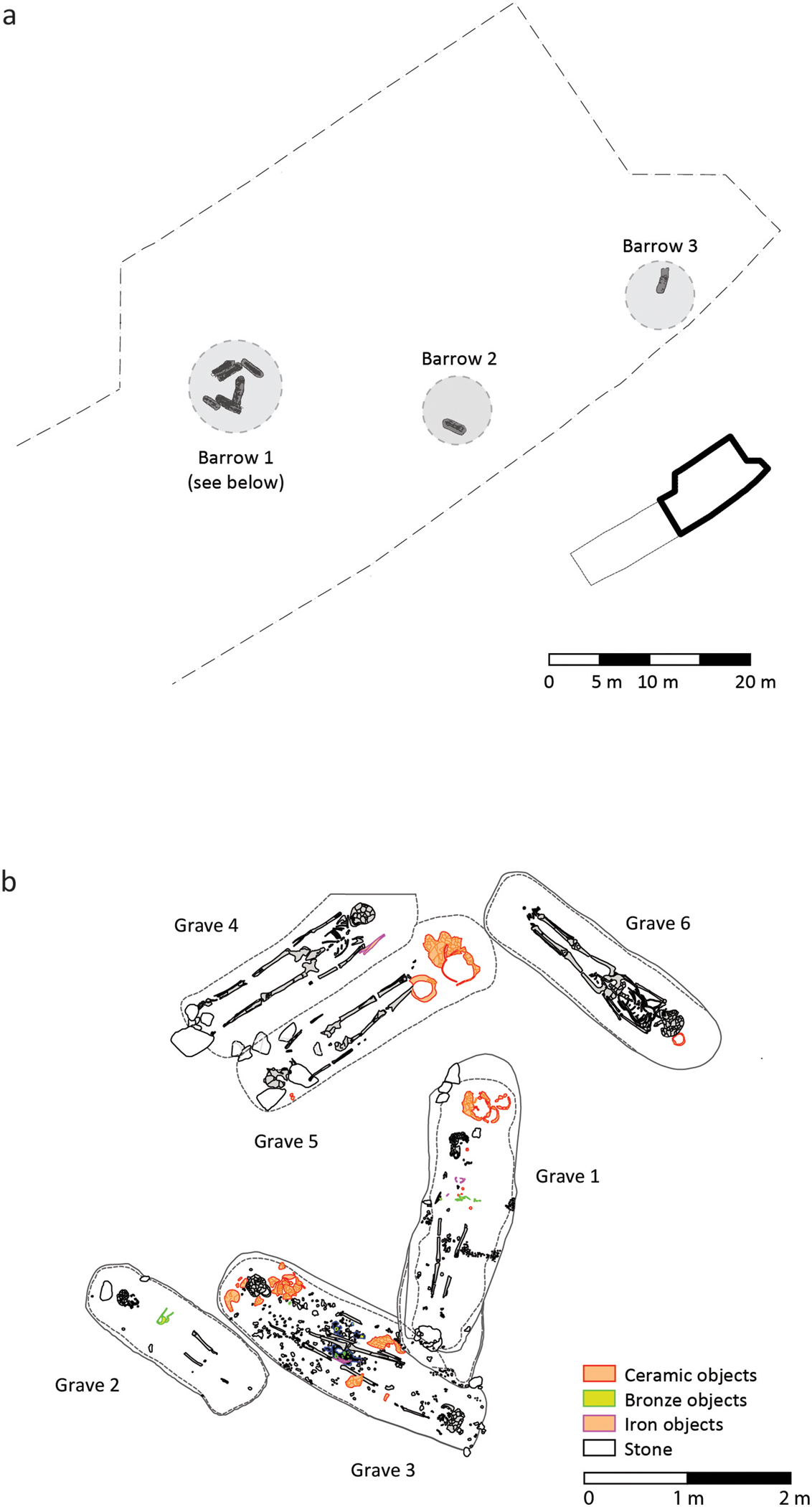
a) Layout of the cemetery, indicating the presumed position of the truncated barrows (after Mason and Mlekuž 2016). b) Plan of the excavated graves within Barrow 1.

Two of the Dolge njive barrows (2 and 3) had been largely destroyed by a combination of Roman settlement activity and medieval agriculture, though both contained at least one inhumation, in each case accompanied by spearheads (Figure 3a; Table 1). Barrow 1, however, was better preserved, covering the remains of six graves containing seven inhumation burials (Figure 3b). All six graves contained extended supine inhumations, including one double burial (Burial 3) comprising two individuals buried head to toe (Table 1). Five of the graves were arranged in a rough circle around the perimeter of the barrow, while the other (Burial 1) lay more centrally. This latter grave, however, cut the edge of Burial 3 and cannot therefore be primary. Although central graves in the region tend to be the earliest within each barrow, there are exceptions in which they belong to the later or even the latest phases of a barrow (e.g. Križ 2019: 277, 293).

The limited evidence for inter-cutting in Barrow 1 (Figure 3b), and the slight degree to which the graves intersect, appears to indicate that the later graves were laid out to respect the earlier ones: this suggests either that the earlier graves were marked on the surface, and/or that the graves were dug over a relatively short period. All of the graves contained grave goods, though the number and composition varied (Table 1); they date on typological grounds mostly to the Stična (I) phase of the Dolenjska Early Iron Age chronology, i.e. Ha C(1). Coupled with stratigraphic and aDNA information (see below), the bodies were most likely deposited over a relatively short period in the early/mid seventh century BC.

## Osteological analysis

The skeletal remains were characterised by cortical exfoliation, root etching and were generally heavily fragmented and incomplete (Nicholls 2017). This was particularly the case for the less dense bones of the axial skeleton (vertebrae, sterna, ribs and ossa coxae) and crania, which hampered osteological assessment of age and sex. The dentition exhibited varying degrees of preservation, but survived most frequently as loose teeth, which permitted age estimation. All remains appear to belong to young (c. 20–35) and middle adults (c. 36–50), and we could tentatively assign sex based on skeletal morphology in only five cases (Table 1). We noted no pathological alterations, although this is unsurprising given the poor condition of the bones.

## Ancient DNA analysis

We successfully analysed aDNA from all nine individuals recovered from Dolge njive; seven from Barrow 1, and single individuals from each of Barrows 2 and 3. The genomic data obtained for these individuals allowed us to determine genetic sex based on the ratio of Y chromosome sequences to combined X and Y chromosome sequences (Table 1), as well as maternal (mitochondrial) and paternal (Y chromosome) lineages (see online Supplementary Information, which also contains information on the population genetics of the group). Regarding the mitochondrial lineages, six of the seven samples from Barrow 1 belong to the H1e5 haplogroup and the seventh carries the H haplogroup. Individuals from Barrows 2 and 3 carry the H5a6 and H1ba haplogroups respectively. All males (across all three barrows) carry a R1b Y chromosome haplogroup, which is one of the major Y chromosome haplogroups in Europe following the Late Neolithic/Bronze Age transition; it became widespread in Europe during the second half of the third millennium BC and is ultimately linked to ancestry from the Eurasian steppe (Allentoft et al. 2015; Haak et al. 2015).

**Figure 4.**
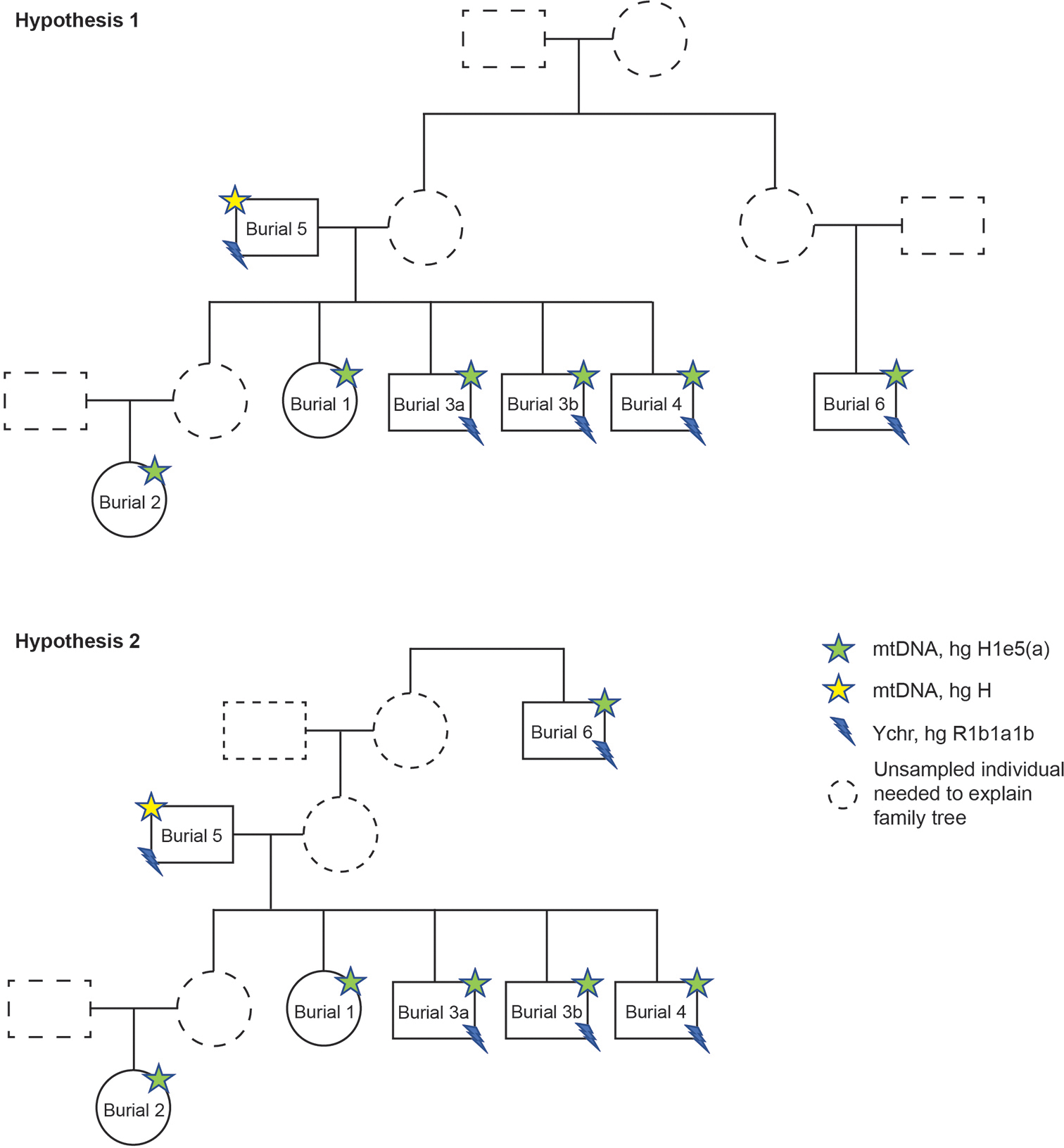
Putative ‘family trees’ based on the aDNA results. In Hypothesis 1, Burial 5 is the father of Burials 1, 3a, 3b and 4, and grandfather of Burial 2. In this scenario, Burial 6 is a maternal cousin of the siblings (Burials 1, 3a, 3b and 4). In Hypothesis 2, Burial 6 is a maternal great-uncle of the siblings, while other relationships remain unchanged.

To explore potential genetic relationships among the Dolge njive individuals, we used the software READ (Monroy-Kuhn et al. 2015). Within Barrow 1, we found that all seven individuals were close biological relatives (Figure 4). Burial 5 represents the father of the individuals in Burials 1, 3a, 3b and 4: three brothers and a sister. The young woman from Burial 2 is a second-degree relative of these siblings and their father. She is most likely, therefore, the granddaughter of the man from Burial 5 and niece of the four siblings. Since she shares her mitochondrial haplogroup with the siblings, it is likely that her mother was a sister of this group. Burial 6 is a third-degree relative of the siblings (Burials 1, 3a, 3b and 4a) with whom he shares the same mitochondrial haplogroup. He may be their maternal cousin, mother’s half-sibling, or great-uncle. Finally, our results highlight that the individuals interred in Barrows 2 and 3 were not close biological relatives either of each other or of the family group buried in Barrow 1.

## Multi-isotope analysis

We analysed multiple isotopes from bones and teeth from all individuals in Barrow 1, examining evidence for diet and mobility (see Supplementary Information). Where possible, we sampled multiple elements from each individual to explore intra-individual isotopic heterogeneity, i.e. lifetime variability. In addition to exploring diet and mobility, the nature of this assemblage provides a rare opportunity to examine the variation in isotope ratios within a familial group.

**Figure 5.**
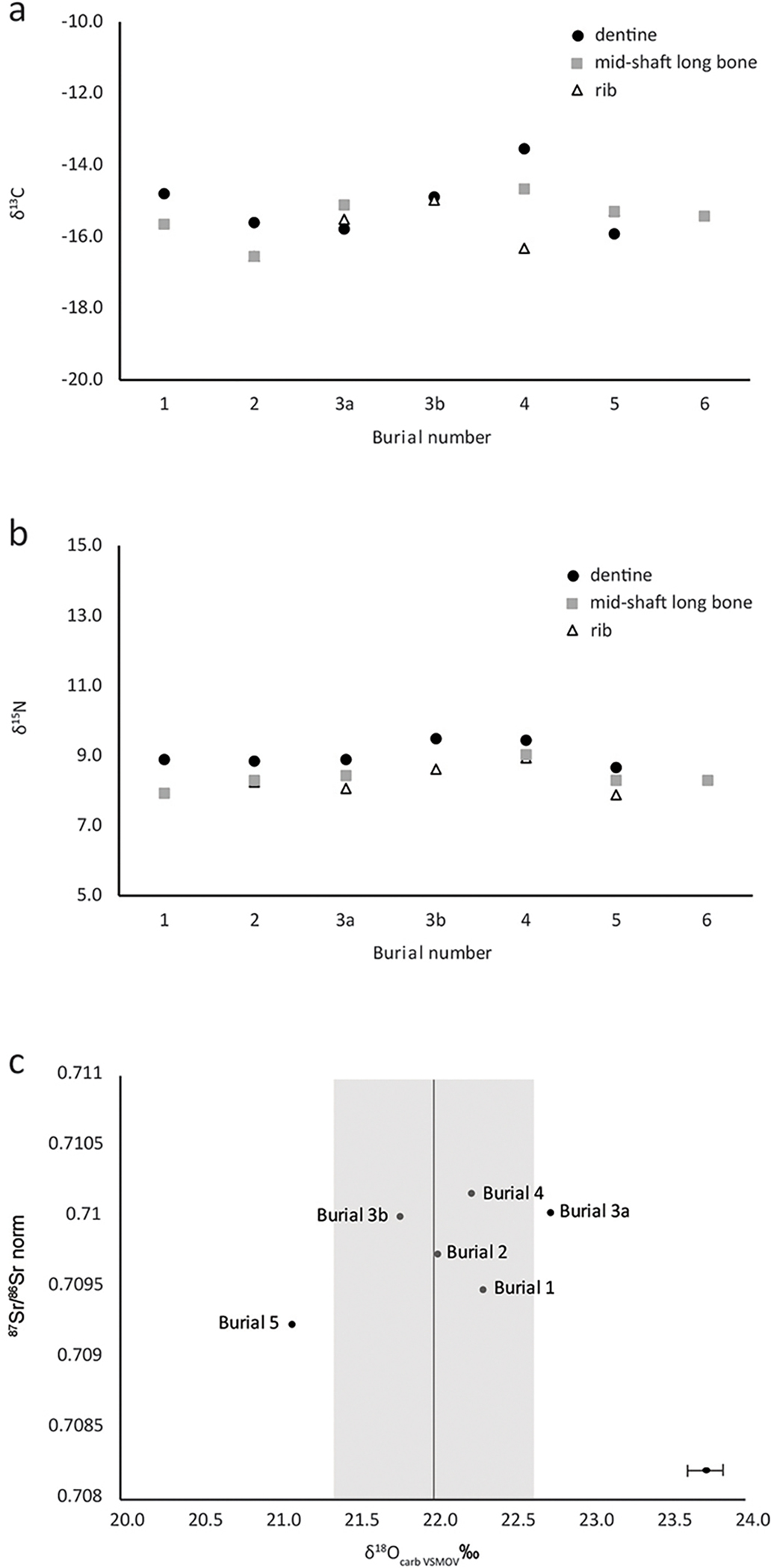
a) Carbon isotope ratios from individuals in Barrow 1. b) Nitrogen isotope ratios from individuals in Barrow 1. c) Carbonate oxygen isotope ratios against strontium isotope ratios obtained from the tooth enamel. Analytical precision based on instrumental error of ± 0.2‰ (too small to be visible on the chart).

The δ^13^C and δ^15^N isotope ratios for the group range from −16.6‰ to −13.6‰ and 7.9‰ to 9.5‰ respectively, indicating a terrestrial-based diet, composed of a mixture of C_3_ and C_4_ plants with some herbivorous animal protein (Tykot 2004; Hedges & Reynard 2007). Values from different elements from each individual show little variation (see Figure 5), indicating consumption of similar sources of terrestrial protein throughout their lives. Δ δ^13^C_CARB-COL_ values consistently exceed 4‰, reflecting a diet high in C_4_ carbohydrates, likely millet (Supplementary Table 1; cf. Lightfoot et al. 2013). This is consistent with the limited previous isotopic analyses for this period in Slovenia (Nicholls et al. 2020; Murray & Schoeninger 1988) and contemporary botanical evidence (Dular & Tecco-Hvala 2007).

**Figure 6.**
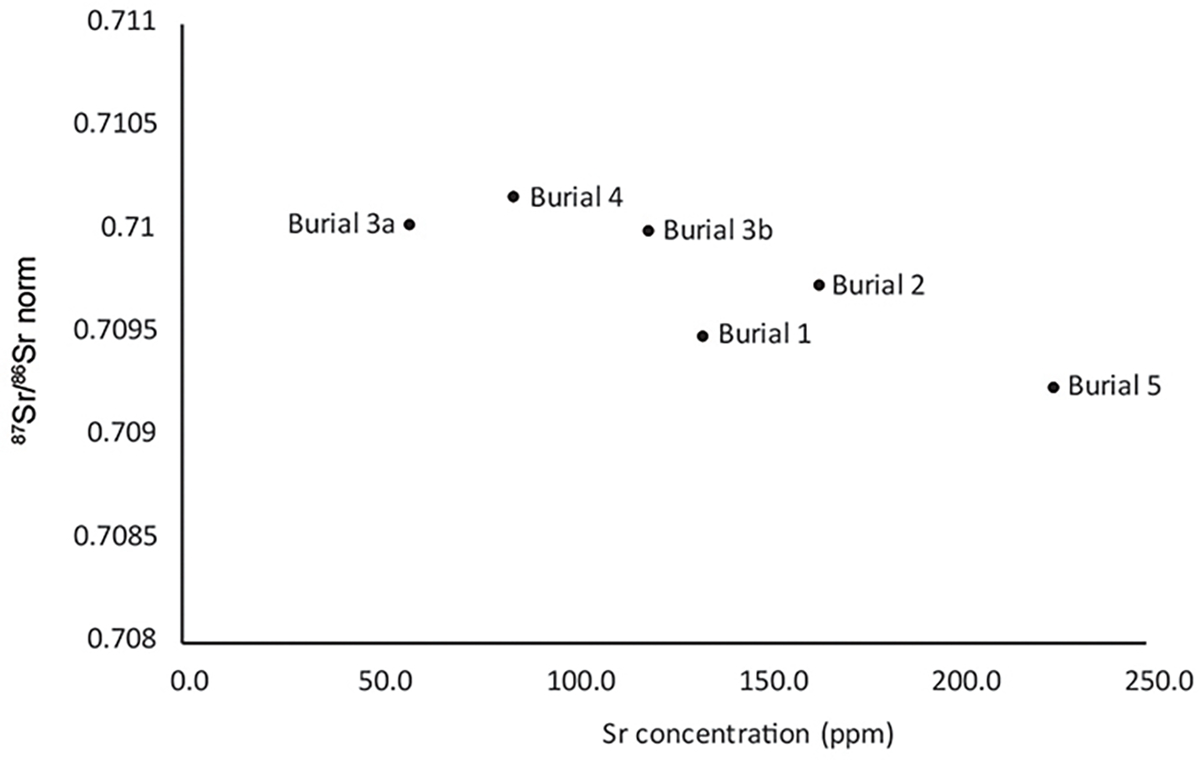
^87^Sr/^86^Sr plotted against strontium concentration for individuals in Barrow 1.

Strontium results are variable, with ^87^Sr/^86^Sr ranging from 0.7091 to 0.7102, and concentrations from 59 to 226 ppm (Supplementary Table 3; Figures 5b and 6). The δ^18^O_CARB_ isotope ratios occupy a relatively narrow range of 21.1‰ to 22.8‰. The ^86^Sr/^87^Sr values are consistent with the variable local geology with no evidence for childhood mobility.

Strontium concentrations may reflect the trophic level of food consumed by an individual, decreasing with increasing trophic level (Evans et al. 2006). The wide variation in strontium concentrations here (Supplementary Table 3; Figure 6) suggests that two of the brothers (Burials 3a and 4) consumed more meat and dairy than the rest, while their father (Burial 5), exhibits by far the highest Sr concentration and thus appears to have eaten more lower trophic level food. This is consistent with the latter’s δ^15^N values, which are the lowest in both dentine and rib collagen. The tooth enamel sampled for this analysis reflects the Sr, O and C_CARB_ isotopic compositions of food consumed during the formation of the tooth crown in early childhood. It appears, therefore, that the father (Burial 5) had a different childhood diet and/or location to his children.

## Sex identification from aDNA, osteology and grave goods

There has been much criticism regarding the traditional methodological basis of ascribing sex based on grave goods, particularly in the absence of secure osteological identification (e.g. Arnold 1996). For the Early Iron Age Dolenjska region, however, Teržan (1985) has proposed that gendered grave goods can be a reliable guide to the biological sex of the interred individual. It is therefore useful to consider how the sex estimations provided by osteological analysis and aDNA analysis compare with those based on grave goods.

Based on artefact typology, it was possible to attribute gender and therefore infer biological sex of six of the individuals interred at Dolge njive (Table 1). In Barrow 1, two burials (1 and 2) were associated with distinct female attire. The Libna-style belt from Burial 3 is characteristic of typologically male graves (Guštin & Preložnik 2005): since this grave contained two burials (the belt seems to have tied both bodies together), it was suggested that at least one is male. The presence of two iron spearheads in Burial 4 similarly suggests that this individual is male. Spearheads also accompanied both individuals identified in the other two (damaged) barrows, suggesting that these individuals are also male. Given the poor condition of the skeletal remains, osteological analysis could contribute little to the sexing of these individuals, providing only tentative sex estimations which proved unreliable (i.e. incorrect when compared with the aDNA results in three of five cases: Table 1). All six attributions based on grave goods, however, are independently confirmed by aDNA analysis.

## AMS dating

**Figure 7.**
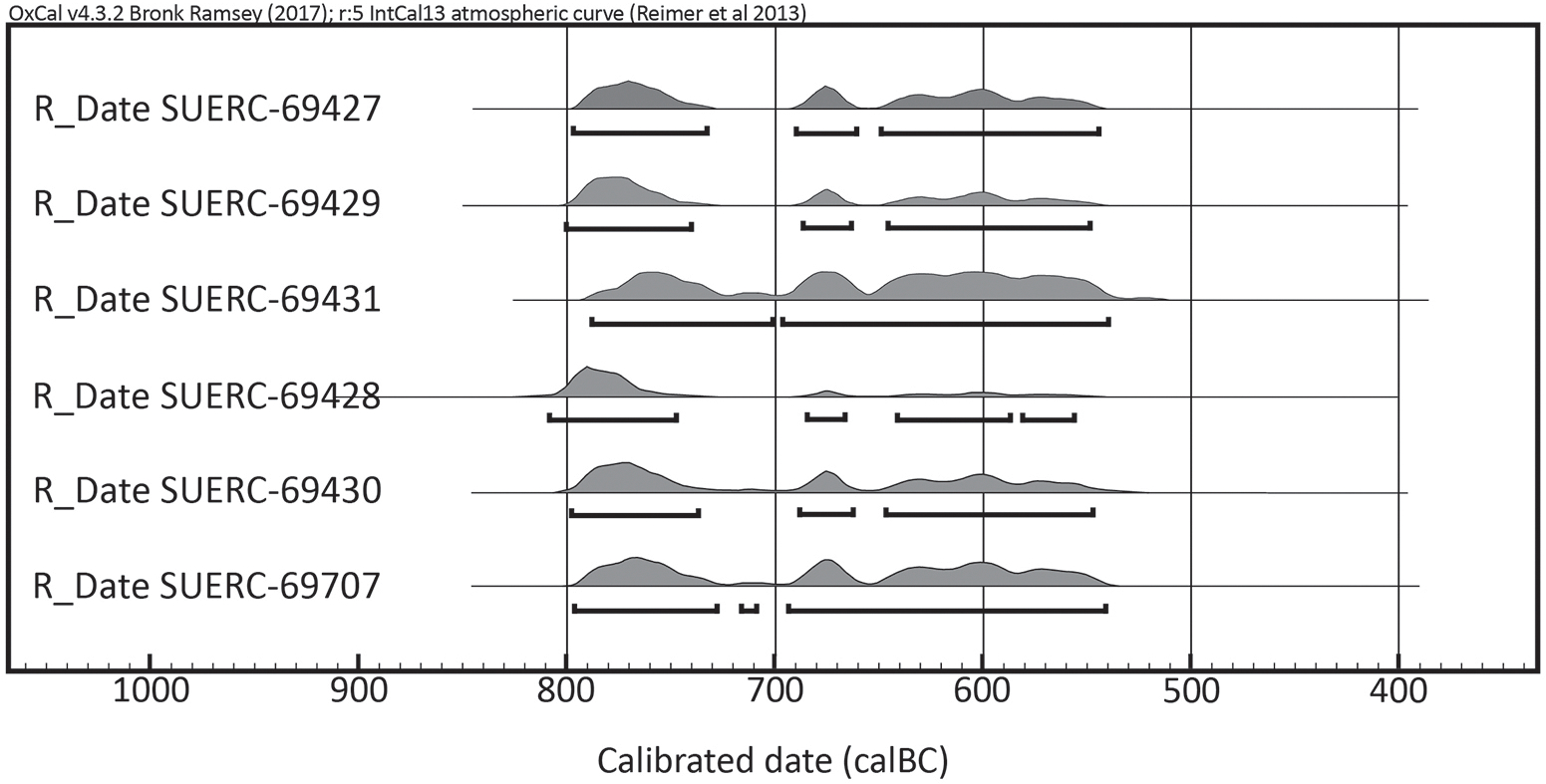
AMS dates from Barrow 1, plotted using OxCal 4.4 (Bronk Ramsey 2009) and IntCal 2020 (Reimer et al. 2020).

Six AMS dates were obtained from the skeletal remains: one from each of the graves in Barrow 1 (Table 2; Figure 7). The results form a consistent series in the period c. 800–540 cal BC. The radiocarbon calibration problems associated with the Hallstatt plateau, however, are such that the AMS dates remain highly imprecise and not readily amenable to further resolution through Bayesian analysis. They are nonetheless consistent with the typological dates for the grave goods, which suggest deposition in the first half or the middle of the seventh century BC.

## Discussion

One of the most promising avenues for ancient DNA research is its potential to reveal patterns of biological relatedness that can inform the analysis of kinship in prehistoric populations (e.g. Sjögren et al. 2020; Fowler et al. 2022). Perhaps the most striking aspect of the present study has been the discovery that all seven individuals buried in Barrow 1 are close biological relatives. The group comprises a father (Burial 5), four of his children (three brothers (Burials 3a, 3b and 4) and a sister (Burial 1)), his granddaughter (Burial 2), and a third-degree male relative of the siblings (Burial 6) who is most likely their maternal cousin, great uncle or mother’s half-brother. Since no biological relationships can be identified between individuals buried in different barrows, it thus seems probable that each accommodated the remains of a distinct familial group, although we should bear in mind that forms of kinship based on factors independent of biological relatedness (e.g. Brück & Frieman 2021) will always elude detection by DNA analysis. The presence in Barrow 1 of four full siblings, and the implied existence of a fifth (mother of Burial 2), together with the absence of definite half-siblings, is also suggestive of a monogamous family structure, though it is always possible that half-siblings may have existed but simply not have been buried within the barrow.

Assessing how this familial structure might articulate with broader kinship patterns requires a dialogue between genetic, ethnographic and archaeological evidence. At first sight, the genetic evidence appears to hint at a possible matrilineal structure in Barrow 1, since all but the father share the same mitochondrial DNA, Burial 2 is related to the rest of the group through the ‘missing’ sister, and Burial 6 is related to the rest of the group through the ‘missing’ mother. Nonetheless, there are several indications that the group composition of Barrow 1 cannot be characterized as being based on matrilineal descent. Most significant, perhaps, is the presence of the biological father and his children, who together form the ‘core family’ within the tomb. This is not typical of matrilineal systems, where the father is typically buried separately with his natal matriline (cf. Ensor et al. 2017: 742). The two female relatives (the mother of the siblings and the mother of the grandchild (Burial 2)), who are the only multi-generational links connecting this core family to the non-first-degree relatives and would thus be central to any scenario of matriliny, are also the only core members of the family that are absent from the tomb. Indeed, it is striking that no individual within Barrow 1 is buried with either their mother or any other maternal ancestor, undermining the notion of a matrilineal basis for selection. Neither does the genetic evidence lend support to the idea of bilateral descent, where spouses are characteristically buried together (Ensor et al. 2017: 741). The physically present individuals within Barrow 1 have a definite male-centred aspect, since they include a father (Burial 5), his four children, and his grandchild. Yet the presence of a maternal relative (Burial 6) and a grandchild from a different patrilineage (Burial 2) suggest that this does not represent a straightforwardly patrilineal system. Despite the close biological relationships between all occupants of Barrow 1, therefore, the genetic evidence does not provide clear support for any specific kinship structure drawn from the ethnographic literature.

In considering these findings, it is important to remember that biological relatedness need not equate to social relatedness, which can be constituted very differently (cf. Brück 2021; Brück & Frieman 2021). In a society where many would have died young, as is evident from the age profile of Barrow 1 itself, many children would have been raised in households of relatives who were not their biological parents. Indeed, relationships of fosterage were apparently institutionalised in certain Iron Age societies in Europe (Karl 2005). In this context, it is possible that the granddaughter and likely cousin from Barrow 1 (Burials 2 and 6) were additional dependents of the senior male (Burial 5), acquiring membership of the patriline through adoption or fosterage. Given their age profile, they may perhaps be dependents who died before marriage, still resident in their ‘father’s’ household. Such a ‘messy’ and fluid, but essentially patrilineal, system could explain the absence from Barrow 1 of the mother, who may have been returned to her natal group for burial (cf. Ensor et al. 2017), although it does not explain the absence of the fifth sibling (the mother of the young woman in Burial 2), who by the same logic would have been buried with her father in Barrow 1. We must also bear in mind that, while societies may espouse certain idealised kinship structures and social practices, these need not always be rigidly adhered to in practice (e.g. Ilcan 1994).

The biological relationships between the individuals buried in Barrow 1, combined with the stratigraphic and osteological evidence, suggests a short use-life for the barrow. The father is identified osteologically as a middle adult, approximately 35–50 years at death (although likely at the older end of this range), while the others (where age can be estimated) died as younger adults. The time gap between their deaths is thus likely to be short, perhaps no more than a decade or so. The internal stratigraphy of Barrow 1 demonstrates that the father’s grave (Burial 5) was cut by that of one of his sons (Burial 4) and by that of the likely cousin (Burial 6) (Figure 3a). The degree of intercutting is, however, minimal and the excavation plan suggests that the latter two graves were laid out to respect that of the father, which was thus probably marked above-ground. Burial 3, which represents the double grave of two brothers (presumably buried at the same time), is cut by that of their sister (Burial 1) and closely respects the grave of their niece (Burial 2). The central placement of Burial 1, combined with its stratigraphic position, suggests that it represents a conscious ‘closing’ of the monument.

The results from Dolge njive have implications for the interpretation of the many other small barrows found individually, in small groups, or within more extensive barrow cemeteries throughout the region. At Kapiteljska njiva, Novo mesto, for example, most of the 67 barrows contained 10 graves or less, and are likely to be of short duration (e.g. Križ 2019: 261, 314–15). It seems likely that the burial groups within these barrows were constituted along similar lines to Barrow 1 at Dolge njive, and it remains unclear why such tombs should be so short-lived, rather than containing multiple generations of the same kinship group. One possibility may be that this society was highly mobile, with kin groups frequently fissioning and moving, leading to the establishment of new burial mounds. Other barrows in the region, however, were used for several centuries and contain much larger numbers of burials; perhaps as many as 400 at Preloge near Magdalenska gora (Dular & Tecco Hvala 2007: 123–6; Tecco Hvala et al. 2004: 124).

Finally, it is important to note the overall sex bias of the Dolge njive group, where males outnumber females by 7:2 (5:2 in the better-preserved Barrow 1). Although based on a small sample, so not statistically significant (P=0.45 for a two-sided test of equal numbers of males and female considering all burials together), this imbalance may suggest that factors other than kinship, perhaps relating to biological sex, social position, or circumstances of death, played a part in determining the composition of the burial population. This of course should not be surprising, given that funerary rites seldom present a straightforward reflection of social organisation, but are rather the outcome of complex processes of negotiation among the living (e.g. Parker Pearson 1999; Fowler 2013).

## Conclusion

The genetic results from Dolge njive confirm the close biological relatedness of individuals buried within the same barrow. They do not, however, provide a definitive guide to understanding the kinship practices of the interred community. While links through the maternal line appear to have been important, the group composition within Barrow 1 does not suggest either a matrilineal or bilateral kinship structure. Evidence for a straightforwardly patrilineal system is also weak, although these results may nonetheless be the products of a more flexible patrilineal system, which pragmatically included the adoption or fosterage of cognatic relatives (including through the female line).

The results from Dolge njive have implications for our wider understanding of Early Iron Age kinship and funerary practices in southeast Europe. The shift from flat cremation cemeteries to the burial of multiple individuals under substantial barrows in itself marks a new concern with the grouping and ordering of the dead and with their visibility in the landscape. Whilst the provision of weaponry and other martial accoutrements in a substantial number of male burials across the region frequently presents the deceased as a physically dominant warrior, this dimension of social power is balanced by female grave assemblages containing objects of considerable intrinsic value and prestige. As at Dolge njive, grave goods were evidently highly gendered, but not necessarily ranked (e.g. Teržan 1985; 2010). The genetic data too suggest the dual importance of male and female descent in determining the composition of the cemetery population. As we have seen in other contexts (Fowler et al. 2022), aDNA analysis reveals kinship structures that are potentially highly complex and unlikely to be reducible to simple patterns of patrilineal or matrilineal descent. Indeed, such kinship relations, built through both male and female lines, will have provided wider and potentially more robust social networks than those based on purely patrilineal or matrilineal principles alone. Future genetic and isotopic analysis, using a large sample of additional barrows from some of the more extensive cemeteries in the region will help us to better understand this complex emerging picture.

## Supplementary Material

Supplementary Information with Tables

Figure S2

Figure S3

Figure S1

## Figures and Tables

**Table 1:** Burials from Dolge

Burial	Grave goods	Stratigraphic relationships	Age	Sex: osteo	Sex: grave goods	Sex: DNA	DNA note
**Barrow 1**							
1	3 pottery vessels, bronze boat fibula with iron pin bearing 4 triangular pendants, iron two-looped semilunate fibula, 3 spindle whorls, 5 amber beads, 2 tin bracelets	Cuts G3	young adult	U	F	F	sibling
2	large & small bronze boat fibulae, amber bead necklace, 2 bronze bracelets and a ringlet	None	young/middle adult	U	F	F	2 deg. rel.
3a	5 pottery vessels, bronze ring and bead belt set of Libna type, iron knife, awl	Cut by G1	young adult	F?	M	M	sibling
3b	*as above*	*as above*	young adult	U	U	M	sibling
4	2 spearheads	Cuts G5	young adult	M?	M	M	sibling
5	3 pottery vessels	Cut by G4 & 6	middle adult	F?	U	M	father
6	pottery vessel	Cuts G5	young adult	M?	U	M	3 deg. rel.
**Barrow 2**							
1	spearhead	None	adult	F?	M	M	unrelated
**Barrow 3**							
1	spearhead	None	unknown	U	M	M	unrelated

**Table 2:** AMS dates from Barrow 1: all dates on human bone

Burial	Lab. code	Raw date (BP)	AMS (95.4% confidence, cal BC)
1	SUERC-69427	2531±29	797–545
2	SUERC-69429	2544±29	800–549
3a	SUERC-69431	2507±29	789–540
4a	SUERC-69428	2569±30	809–557
5	SUERC-69430	2537±29	798–548
6	SUERC-69707	2525±31	796–542
