# Supplementary material for: Kinship practices in Early Iron Age southeast Europe: genetic and isotopic analysis of burials from the Dolge njive barrow cemetery, Dolenjska, Slovenia
Source: Antiquity. Author manuscript; Available in PMC 2024 Nov 24. (PMC11586097; doi:10.15184/aqy.2023.2)
Supplement: Supplementary Information with Tables [file NIHMS2033570-supplement-Supplementary_Information_with_Tables.docx]

**[Supplementary material]**

**Kinship practices in Early Iron Age southeast Europe: genetic and isotopic analysis of burials from Dolge njive, Dolenjska, Slovenia**

**Ancient DNA analysis: technical report**

***Introduction***

All of the surviving burials from the barrow cemetery at Dolge njive were analysed for aDNA, comprising seven individuals from Barrow 1 and one individual each from Barrows 2 and 3. Analysis was undertaken at the Harvard Medical School ancient DNA facilities. Powdered samples were obtained in a clean dedicated room. DNA was extracted using a method that is optimised to retain small DNA fragments (Dabney *et al*. 2013; Rohland *et al*. 2018). Between one and four double-stranded libraries were built for each sample with the enzyme Uracil-DNA Glycosylase (UDG) to remove characteristic ancient DNA damage and thereby greatly reduce the rate of damage-induced errors (Rohland *et al*. 2015). Libraries were enriched both for sequences overlapping mitochondrial DNA (Fu *et al*. 2013) and for sequences overlapping about 1.2 million nuclear targets (Fu *et al*. 2015; Mathieson *et al*. 2015), and then sequenced on Illumina instruments. The paired-end reads obtained from sequencing were merged and aligned to the mitochondrial DNA reference sequence RSRS (Behar *et al*. 2012) and human genome reference sequence (version hg19) using BWA (version 0.6.1) (Li & Durbin 2010), following previously published protocols (e.g. Mathieson *et al*. 2015). Mitochondrial haplogroups were determined based on the consensus sequence using Haplogrep2 (Weissensteiner *et al*. 2016). We assessed ancient DNA authenticity based on consistency with the mitochondrial DNA consensus using contamMix (Fu *et al*. 2013) and to the consensus sequence on the X chromosome in males using ANGSD. For population genetic analysis, we represented each targeted single nucleotide polymorphism (SNP) by a randomly selected sequence (for SNPs covered at least once).

Table S1 presents the results by individual libraries, while Table S2 presents the results by sample (where we merge data for multiple libraries).

***Kinship analysis***

We performed kinship analysis, using the software READ (Relationship Estimation from Ancient DNA; Kuhn *et al*. 2018). Our results highlight several biological relationships among the individuals from Barrow 1:

First degree relatives:

- I22935 and I5686
- I22935 and I5685
- I22935 and I5684
- I22935 and I22934
- I5684 and I5686
- I5684 and I5685
- I5685 and I5686
- I22934 and I5686
- I22934 and I5684
- I22934 and I5685

Second degree relatives:

- I23971 and I5685
- I23971 and I5684
- I23971 and I5686
- I23971 and I22934
- I23971 and I22935

Third degree relatives:

- I5687 and I5685
- I5687 and I5686
- I5687 and I5684
- I5687 and I22935

***Phenotypes***

We used the HIrisPlex SNPs panel (Walsh *et al*. 2013; Chaitanya *et al*. 2018), which analyses 41 SNPs, to predict eye and hair colour, and skin pigmentation.

We were able to predict eye colour for four individuals:

- I5684 has a substantial probability of having blue eyes (76%)
- I5685 has a substantial probability of having blue eyes (64%)
- I5686 has a substantial probability of having blue eyes (76%)
- I5687 has a substantial probability of having blue eyes (78%)

It was not possible to predict hair or skin colour with any confidence due to the absence of data from too many essential SNPs.

***Population Genomics Analysis***

In order to carry out population genomics analysis we used the database provided by the David Reich Laboratory (which curates previously published data from many sources), and which is available at <https://reich.hms.harvard.edu/allen-ancient-dna-resource-aadr-downloadable-genotypes-present-day-and-ancient-dna-data>

The comparative dataset comprises different types of data based on capture and shotgun sequencing. The shotgun sequencing data are in many cases derived from low coverage genomes (from 0.1 to 0.5X). This means that the recovered sequences are randomly distributed across the genome and not targeted as in capture analysis.

Of the first degree relatives, we only use individual I5685 for the following analyses (except the PCA), as this individual has the best coverage of the group comprising I5685, I5687, I23971, I22933 and I22936.

*Principal Component Analysis*

The PCA is built by projecting the ancient DNA data onto a set of modern European and Near Eastern populations. Here we project available data for Iron Age populations in Western Europe (Gamba *et al*. 2014; Allentoft *et al*. 2015; Schiffels *et al*. 2016; Martiniano *et al*. 2016; Damgaard *et al*. 2018; Mathieson *et al*. 2018; Antonio *et al*. 2019; Jarve *et al*. 2019; Olalde *et al*. 2019; Saag *et al*. 2019; Sikora *et al*. 2019; Brunel *et al*. 2020; Fernandes *et al*. 2020; Marcus *et al*. 2020; Margaryan *et al*. 2020).

Our samples from Dolge njive, as well as others samples from Slovenia, plot in an intermediate position between Western European samples (UK, France, Spain) and Southern European samples (Italy) (Figure S1). However, one individual (I22936) seems to have more affinity with Southern European samples (Italy, Eastern Mediterranean or Punic samples from Sardinia), but this outlying position potentially reflects the limited coverage (24864 SNPs among the 600 000 SNPs targeted).

*Affinities among the Dolge njive samples*

We performed a qpWave analysis as implemented in ADMIXTOOLS (Patterson *et al*. 2012) to determine if individuals from Dolge njive can be defined as a clade, following Fernandes *et al*. 2020. For this test, we used the following set of populations: Mbuti.SDG, EHG, Russia_Afanasievo, Turkey_N, Germany_EN_LBK, WHG, Russia_Samara_EBA_Yamnaya. Outliers are defined when p < 0.1.

Our results highlight that all individuals from the Dolge njive site form a clade, including individual I22936 who is an apparent outlier on the PCA (and who was buried in a separate barrow from the biologically related individuals in Barrow 1). This is consistent with their outlying position in the PCA being due to limited data size.

*Affinities with ancestral populations*

We performed a qpAdm analysis as implemented in ADMIXTOOLS (Haak *et al*. 2015) to investigate the percentage of several ancestries in the individuals from Dolge njive. Here we estimate the relative proportions of EEF, Yamnaya (Steppe) and Western Hunter Gatherer ancestries. We performed this test on each sample.

Our results indicate that individuals from Dolge njive seem quite homogeneous in terms of ancestry (Figure S2; note that two individuals (I22933 and I23971) are not plotted as the model does not fit (p value < 0.05)).

*Affinities with contemporaneous populations*

We computed an outgroup-f3 statistic (Raghavan *et al*. 2014) as implemented in ADMIXTOOLS in the form (Mbuti; Individual from Dolge njive, Iron Age*) where we tested the individuals from Dolge njive against the available Iron Age groups. This statistic allows us to explore the affinities between two groups: a high f3 means that the groups are close and a low f3 means that the groups show few affinities.

Our results show that the individuals from Dolge njive are closer to other samples from Slovenia than from other parts of Europe, with the exception of two outliers: one from Spain and one from England.

***Data Availability***

The raw data are available as aligned sequences (bam files) through the European Nucleotide Archive under accession number PRJEBXXXXX **[to be completed on acceptance]**

**Multi-isotope analysis**

Isotope analyses were conducted on the bones and teeth of the individuals buried in Barrow 1 to explore evidence of heterogeneity within the diet and potential differences in geographical location within the group during their childhoods (Table S3).

***Dietary Reconstruction***

Carbon (δ^13^C) and nitrogen (δ^15^N) collagen isotope analyses are commonly used to reconstruct the protein contribution to the diet (Ambrose & Norr 1993; Keller & Schoeninger 2007). Nitrogen isotopes track nitrogen pathways within the local ecosystem, reflect the trophic level of the consumer, and detect whether the diet is composed of primarily terrestrial or aquatic resources (Hedges & Reynard 2007; Schoeninger *et al*. 1983). Carbon isotopes track carbon pathways through plants into the food chain reflecting the contribution of C3 or C4 vegetation within the diet and can also be used as a means of differentiating between terrestrial and marine food chains (Lee-Thorp *et al*. 1989; Richards & Hedges 1999; Richards 2003; Tafuri *et al*. 2009). Carbonate carbon isotopes (δ^13^C_CARB_), which are extracted from the mineral component, can further elucidate the carbon component of the diet, as, unlike carbon isotopes from bone and dentine collagen, δ^13^C_CARB_ reflects the whole diet, including carbohydrates and lipids (Katzenberg & Waters-Rist 2018; Loftus & Sealy 2012). For an overview of the principles behind dietary reconstruction using stable isotopes and reviews of previous research within this field see Makarewicz & Sealy 2015, Hedges & Reynard 2007, and Katzenburg & Waters-Rist 2018.

By sampling skeletal elements, with different turnover rates, within the same skeleton it is possible to explore variations in diet over an individual’s lifetime (Reitsema & Vercellotti 2012). Once formed, teeth do not undergo significant remodelling and so preserve isotopic information on dietary input during the formation of the tooth, i.e. during childhood and adolescence (Beaumont *et al*. 2013; Fuller *et al*. 2003). The mid-shaft femur gradually remodels over an individual’s lifetime and thus isotope values represent a much longer period, reflecting an average protein consumption throughout adulthood (Hedges *et al*. 2007). The rib collagen is constantly remodelling (up to 7.7% a year for adults), and so will reflect a relatively short period of dietary consumption prior to death (Parfitt 2002).

Carbon and nitrogen stable isotope data were produced from the bulk collagen of long bones (principally mid-shaft femur), ribs and dentine (principally permanent first molars; dentine samples were removed from the apex (c. final 2mm) of tooth roots, reflecting the isotopic composition of diet and metabolic condition of an individual in the final stages of the tooth’s development (i.e. late childhood/early adolescence)). Where available, all three elements were sampled from each skeleton, as a means of detecting inter and intra-individual isotopic heterogeneity. All samples included in this study produced a >1% collagen yield and had C:N ratios between 2.9 and 3.5, indicative of well-preserved collagen (Van-Klinken 1999). Bone and dentine collagen was extracted using a modified Longin method (Longin 1971; Brown *et al*. 1988). The isotopic analyses were carried out at the University of Bradford Light Stable Isotope Facility using the protocol outlined in Nicholls *et al*. (2020).

To explore the subtle differences in individual δ^13^C isotope ratios further, dental enamel samples were taken for carbonate analysis (France & Owsley 2015). Enamel samples rather than bone apatite was chosen to minimise potential diagenetic contamination (Snoeck *et al*. 2015). For both the δ^13^C_CARB_ and δ^18^O_CARB_ analyses, tooth enamel was removed using dental burs and these were taken from the same teeth as those sampled for dentine collagen. The resultant powder was then agitated in NaOCl using a whirl mixer and then left for 30 minutes, to remove any remaining organic material. Samples were then centrifuged and rinsed 3 times in deionised water, before an acetic acid solution was added and the samples left for 10 minutes to remove any diagenetic carbonate. A further 4 rinses were carried out with deionised water before each sample was frozen and freeze dried. ~2mg of enamel powder was reacted with 100% phosphoric acid at 70°C for 60 minutes in a Thermo Delta V connected to a Gasbench II, to produce CO2. The precision of this analysis is also ±0.2. The analysis was undertaken at the University of Bradford Light Stable Isotope Facility. Internal standards were run alongside samples to monitor measurement variation. These Internal standards were Merck (pure calcium carbonate, carbon: -34.45, oxygen: +13.35 VSMOW) and OES (ostrich eggshell, carbon: -10.72, oxygen: +25.45 VSMOW).

***Childhood location and mobility***

Strontium and oxygen isotopes vary depending on the local geology and climate (Britton *et al*. 2009; Bentley & Knipper 2005; Montgomery 2010). Carbonate oxygen isotopes (δ^18^O _CARB_) largely reflect local water sources derived from meteoric water (rain and snow), or from recycled water (lakes, wells or springs) (Bentley & Knipper 2005; Sponheimer & Lee-Thorp 1999). Isotope ratios of strontium directly represent the geographic area of food production/acquisition and, thus, the mobility of dietary resources or their consumers, or both (Budd *et al*. 2000; Montgomery *et al*. 2007). They are dependent upon the variation in strontium isotope composition of local rocks and geology, with the addition of local rainfall and terrestrial aerosols (Budd *et al*. 2000; Montgomery *et al*. 2007; Montgomery 2010). Thus, analyses of these isotopes in dental enamel, which does not remodel, can be used as a means of determining childhood origins and residential mobility that may have occurred during formation of the tooth (Evans *et al*. 2006; Prowse *et al*. 2007).

Carbonate oxygen isotopes (δ^18^O _CARB_) were measured alongside carbonate carbon (δ^12^C _CARB_) using the method outlined above. Strontium analysis was undertaken at the Durham University Earth Sciences Department using the protocol outlined in Gron *et al*. (2016).

The area surrounding the Dolge njive cemetery, which lies approximately 155m above sea level, is geologically complex. If the interred individuals inhabited the hillfort, at least four different geological formations surrounded them. These include marls, sandstones and quartzite marls (Miocene Age); limestones and calcirudite (Miocene Age); coarse-grained dolomite (Triassic); and other marls and limestones (Cretaceous) (Dular and Tecco Hvala 2007: 44–64; Geološki zavod Slovenije/Geological Survey of Slovenia). There are also Quaternary and Holocene clay deposits to the south of the barrows in the vicinity of Dolge njive itself, derived from erosion products from the Triassic and Cretaceous deposits (as above) in the Bela Cerkev and Draga areas.

As the geology of the immediate area is so geologically diverse, ^86^Sr/^87^Sr values obtained for individuals buried at Dolge njive probably represent an average of the isotope ratios consistent with these bedrocks and deposits. The ^86^Sr/^87^Sr values of 0.709 – 0.7101 are higher than might be expected from chalk or limestone geologies, and may have been influenced by the fluvial deposits, or could indicate resources coming from further afield (Bentley & Knipper 2005; Evans *et al*. 2006; Montgomery *et al*. 2007; Montgomery 2010). Without any baseline data reflecting potential local values of biologically available strontium (e.g. from plant, soil or faunal samples) and drinking water, it is difficult to speculate further on potential differences in childhood mobility between the individuals from Barrow 1.

**Osteological analysis: methods**

Preservation and completeness were recorded for each skeleton from Dolge njive. Preservation relates to the condition of the bone (which was frequently affected by weathering), post-mortem breaks and loss of the cortical surface. Completeness was recorded as a percentage of the skeleton present for analysis. The preservation and completeness in this population severely impacted the osteological analysis, particularly as the ossa coxae were poorly preserved in this population (Nicholls 2017). As all individuals were adult, no methods for the analysis of non-adults are discussed here.

For adults, biological sex was assessed using the pelvis and skull, using the following traits: greater sciatic notch, pre-auricular sulcus (Walker 2005), ventral arc, sub-pubic concavity, ischiopubic ramus (Phenice 1969; Klales *et al*. 2012), nuchal crest, supra-orbital margin, glabella, mastoid process, mental eminence (Buikstra and Ubelaker 1994; Walker 2008), parietal eminences, posterior zygomatic arch, the shape of the orbits and orbital margins, gonial angle, and presence or absence of gonial flaring (Brothwell 1981: 59–61; İşcan and Steyn 2013: 160–9).

Adult age was estimated using the pubic symphysis, auricular surface, cranial suture closure and dental wear (Brooks & Suchey 1990; Buckberry & Chamberlain 2002; Meindl & Lovejoy 1985; Brothwell 1981). Due to poor levels of preservation, age estimates relied upon dental wear; however, it must be noted that there are no published, calibrated, population-specific standards for Slovenia and there are not enough well-preserved juvenile jaws to calibrate dental wear for the region; thus, age estimates are very approximate. Each individual was placed into an age category for analysis and interpretation (Table S4).

**Concordance with the excavation record**

Table S5 provides a concordance between the burial numbers used in this paper and the context and small finds numbers used in the original excavation archive.

**Supplementary references**

Ambrose, S.H. & Norr, L. 1993. Experimental evidence for the relationship of the carbon isotope ratios of whole diet and dietary protein to those of bone collagen and carbonate, in J. B. Lambert & G. Grupe (eds) *Prehistoric human bone: archaeology at the molecular level*: 1–37. Berlin: Springer.

Allentoft, M. *et al*. 2015. Population genomics of Bronze Age Eurasia. *Nature* 522: 167–72.

Antonio M.L. *et al*. 2019. Ancient Rome: a genetic crossroads of Europe and the Mediterranean. *Science* 366 (6466): 708–14.

Beaumont, J., Gledhill, A., Lee-Thorp, J. & Montgomery, J. 2013. Childhood diet: a closer examination of the evidence from dental tissue using stable isotope analysis of incremental human dentine. *Archaeometry* 55 (2): 277–95.

Behar, D.M. *et al*. 2012. A ‘Copernican’ reassessment of the human mitochondrial DNA tree from its root. *American Journal of Human Genetics* 90: 675–84.

Bentley, R.A. & Knipper, C. 2005. Geographical patterns in biologically available strontium, carbon and oxygen isotope signatures in prehistoric SW Germany. *Archaeometry* 47 (3): 629–44.

Britton, K., Grimes, V., Dau, J. & Richards, M.P. 2009. Reconstructing faunal migrations using intra-tooth sampling and strontium and oxygen isotope analyses: a case study of modern caribou (Rangifer tarandus granti). *Journal of Archaeological Science* 36 (5): 1163–72.

Brooks, S. & Suchey, J.M. 1990. Skeletal age determination based on the os pubis: a comparison of the Acsádi-Nemeskéri and Suchey-Brooks methods. *Human Evolution* 5 (3): 227‒38.

Brothwell, D. 1981. *Digging up bones* (3rd edition)*.* Oxford: Oxford University Press.

Brown, T.A., Nelson, D.E., Vogal, J.S. & Southon, J.R. 1988. Improved collagen extraction by modified Longin method. *Radiocarbon* 30: 171–7.

Brunel, S. *et al*. 2020. Ancient genomes from present-day France unveil 7,000 years of its demographic history. *Proceedings of the National Academy of Sciences* 117 (23): 12791–8.

Buckberry, J.L. & Chamberlain, A.T. 2002. Age estimation from the auricular surface of the ilium: a revised method. *American Journal of Physical Anthropology* 119 (3): 231‒9.

Budd, P., Montgomery, J., Barreiro, B. & Thomas, R.G. 2000. Differential diagenesis of strontium in archaeological human dental tissues. *Applied Geochemistry* 15 (5): 687–94.

Buikstra, J.E. & Ubelaker, D.H. 1994. *Standards for data collection from human skeletal remains.* Arkansas Archeological Survey Research Series. Vol. 44. Fayetteville: Wiley.

Chaitanya, L. *et al*. 2018. The HIrisPlex-S system for eye, hair and skin colour prediction from DNA: introduction and forensic developmental validation. *Forensic Science International: Genetics* 35: 123–35.

Dabney, J. *et al*. 2013. Complete mitochondrial genome sequence of a Middle Pleistocene cave bear reconstructed from ultrashort DNA fragments. *Proceedings of the National Academy of Sciences* 110: 15758–63.

Damgaard, P. de B. *et al*. 2018. 137 ancient human genomes from across the Eurasian steppes. *Nature* 557: 369–74.

Dular, J. & Tecco Hvala, S. 2007. *South-eastern Slovenia in the Early Iron Age: settlement, economy, society/Jugovzhodna Slovenija v starejši železni dobi: poselitev, gospodarstvo, družba*. Opera Instituti Archaeologici Sloveniae 12. Ljubljana: Založba ZRC.

Evans, J.A., Chenery, C.A. & Fitzpatrick, A.P. 2006. Bronze Age childhood migration of individuals near Stonehenge, revealed by strontium and oxygen isotope tooth enamel analysis. *Archaeometry* 48 (2): 309–21.

Fernandes, D.M. *et al*. 2020. The spread of steppe and Iranian-related ancestry in the islands of the western Mediterranean. *Nature Ecology and Evolution* 4: 334–45.

France, C.A. & Owsley, D.W. 2015. Stable carbon and oxygen isotope spacing between bone and tooth collagen and hydroxyapatite in human archaeological remains. *International Journal of Osteoarchaeology* 25 (3): 299–312.

Fu, Q. *et al*. 2013. DNA analysis of an early modern human from Tianyuan Cave, China. *Proceedings of the National Academy of Sciences* 110: 2223–7.

Fu, Q. *et al*. 2015. An early modern human from Romania with a recent Neanderthal ancestor. *Nature* 524: 216–9.

Fuller, B.T., Richards, M.P. & Mays, S.A. 2003. Stable carbon and nitrogen isotope variations in tooth dentine serial sections from Wharram Percy. *Journal of Archaeological Science* 30 (12): 1673–84.

Gamba, C. *et al*. 2014. Genome flux and stasis in a five millennium transect of European prehistory. *Nature Communications* 5: 5257.

Geološki zavod Slovenije/Geological Survey of Slovenia. nd. *Osnovna Geološka karta*. <https://ogk100.geo-zs.si/>. Online source consulted 29/09/2021.

Gron, K.J., Montgomery, J., Nielsen, P.O., Nowell, G.M., Peterkin, J.L., Sørensen, L. & Rowley-Conwy, P. 2016. Strontium isotope evidence of early Funnel Beaker Culture movement of cattle. *Journal of Archaeological Science: Reports* 6: 248–51.

Haak, W. *et al*. 2015. Massive migration from the steppe was a source for Indo-European languages in Europe. *Nature* 522 (7555): 207–11.

Hedges, R.E.M., Clement, J.G., Thomas, C.D.L. & O’Connell, T.C. 2007. Collagen turnover in the adult femoral mid-shaft: modeled from anthropogenic radiocarbon tracer measurements. *American Journal of Physical Anthropology* 133 (2): 808–16.

Hedges, R.E.M. & Reynard, L. 2007. Nitrogen isotopes and the trophic level of humans in archaeology. *Journal of Archaeological Science* 34 (8): 1240–51.

İşcan, M.Y. & Steyn, M. 2013. *The human skeleton in forensic medicine.* Springfield: Charles Thomas.

Järve, M. *et al*. 2019. Shifts in the genetic landscape of the Western Eurasian Steppe associated with the beginning and end of the Scythian dominance. *Current Biology* 29 (14): 2430–41.

Katzenberg, M.A. & Waters‐Rist, A.L. 2018. Stable isotope analysis: a tool for studying past diet, demography, and life history, in M.A. Katzenberg & A.L. Graue (eds) *Biological anthropology of the human skeleton (3^rd^ edition)*: 467–504. New York: Wiley.

Kellner, C.M. & Schoeninger, M.J. 2007. A simple carbon isotope model for reconstructing prehistoric human diet. *American Journal of Physical Anthropology* 133 (4): 1112–27.

Klales, A.R., Ousley, S.D. & Vollner, J.M. 2012. A revised method of sexing the human innominate using Phenice’s nonmetric traits and statistical methods*. American Journal of Physical Anthropology* 149 (1): 104‒14.

Kuhn, J.M.M., Jakobsson, M. & Günther, T. 2018. Estimating genetic kin relationships in prehistoric populations. *PLoS ONE* 13 (4): 1–21.

Lee-Thorp, J.A., Sealy, J.C. & van der Merwe, N.J. 1989. Stable carbon isotope ratio differences between bone collagen and bone apatite, and their relationship to diet. *Journal of Archaeological Science* 16 (6): 585–99.

Li, H. & Durbin, R. 2010. Fast and accurate long-read alignment with Burrows–Wheeler transform. *Bioinformatics* 26: 589–95.

Loftus, E. & Sealy, J. 2012. Interpreting stable carbon isotopes in human tooth enamel: an examination of tissue spacings from South Africa. *American Journal of Physical Anthropology* 147 (3): 499–507.

Longin, R. 1971. New method of collagen extraction for radiocarbon dating. *Nature* 230: 241–2.

Makarewicz, C.A. & Sealy, J. 2015. Dietary reconstruction, mobility, and the analysis of ancient skeletal tissues: expanding the prospects of stable isotope research in archaeology. *Journal of Archaeological Science* 56: 146–58.

Marcus, J.H. *et al*. 2020. Genetic history from the Middle Neolithic to present on the Mediterranean island of Sardinia. *Nature Communications* 11: 939.

Margaryan, A. *et al*. 2020. Population genomics of the Viking world. *Nature* 585: 390–6.

Martiniano, R. *et al*. 2016. Genomic signals of migration and continuity in Britain before the Anglo-Saxons. *Nature Communications* 7 (1): 1–8.

Mathieson, I. *et al*. 2015. Genome-wide patterns of selection in 230 ancient Eurasians. *Nature* 528: 499–503.

Mathieson, I. *et al.* 2018. The genomic history of southeastern Europe. *Nature* 555: 197–203.

Meindl, R.S. & Lovejoy, C.O. 1985. Ectocranial suture closure: a revised method for the determination of skeletal age at death based on the lateral-anterior sutures. *American Journal of Physical Anthropology* 68 (1): 57‒66.

Montgomery, J. 2010. Passports from the past: investigating human dispersals using strontium isotope analysis of tooth enamel. *Annals of Human Biology* 37 (3): 325–46.

Montgomery, J., Evans, J.A. & Cooper, R.E. 2007. Resolving archaeological populations with Sr-isotope mixing models. *Applied Geochemistry* 22 (7): 1502–14.

Nicholls, R.A. 2017. *More than bones. An investigation of life, death and diet in later prehistoric Slovenia and Croatia*. Unpublished doctoral dissertation, University of Bradford.

Nicholls, R., Buckberry, J., Beaumont, J., Črešnar, M., Mason, P., Armit, I. & Koon, H. 2020. A carbon and nitrogen isotopic investigation of a case of probable infantile scurvy (6th–4th centuries BC, Slovenia). *Journal of Archaeological Science: Reports* 30: 102206.

Olalde, I. *et al*. 2019. The genomic history of the Iberian Peninsula over the past 8000 years. *Science* 363 (6432): 1230–4.

Parfitt, A.M. 2002. Misconceptions (2): turnover is always higher in cancellous than in cortical bone. *Bone* 30 (6): 807–9.

Patterson, N., Moorjani, P., Luo, Y., Mallick, S., Rohland, N., Zhan, Y., Genschoreck, T., Webster, T. & Reich, D. 2012. Ancient admixture in human history. *Genetics* 192: 1065–93.

Phenice, T.W. 1969. A newly developed visual method of sexing the os pubis. *American Journal of Physical Anthropology* 30 (2): 297‒301.

Prowse, T.L., Schwarcz, H.P., Garnsey, P., Knyf, M., Macchiarelli, R. & Bondioli, L. 2007. Isotopic evidence for age‐related immigration to imperial Rome. *American Journal of Physical Anthropology* 132 (4): 510–19.

Raghavan, M. 2014. Upper Palaeolithic Siberian genome reveals dual ancestry of Native Americans. *Nature* 505: 87–91.

Reitsema, L.J. & Vercellotti, G. 2012. Stable isotope evidence for sex‐and status‐based variations in diet and life history at medieval Trino Vercellese, Italy. *American Journal of Physical Anthropology* 148 (4): 589–600.

Richards, M.P. 2003. Sharp shift in diet at onset of Neolithic. *Nature. Brief Communications* 425: 366.

Richards, M. P. & Hedges, R. E. 1999. Stable isotope evidence for similarities in the types of marine foods used by Late Mesolithic humans at sites along the Atlantic coast of Europe. *Journal of Archaeological Science* 26 (6): 717–22.

Rohland, N., Glocke, I., Aximu-Petri, A., & Meyer, M. 2018. Extraction of highly degraded DNA from ancient bones, teeth and sediments for high-throughput sequencing. *Nature Protocols* 13: 2447–61.

Rohland, N., Harney, E., Mallick, S., Nordenfelt, S. & Reich, D. 2015. Partial uracil-DNA-glycosylase treatment for screening of ancient DNA. *Philosophical Transactions of the Royal Society of London B Biological Sciences* 370: 20130624.

Saag, L. *et al*. 2019. The arrival of Siberian ancestry connecting the Eastern Baltic to Uralic speakers further east. *Current Biology* 29 (10): 1701–11.

Schiffels, S. *et al*. 2016. Iron Age and Anglo-Saxon genomes from East England reveal British migration history. *Nature Communications* 7 (1): 1–9.

Schoeninger, M.J., DeNiro, M.J. & Tauber, H. 1983. Stable nitrogen isotope ratios of bone collagen reflect marine and terrestrial components of prehistoric human diet. *Science* 220 (4604): 1381–3.

Sikora, M. *et al*. 2019. The population history of northeastern Siberia since the Pleistocene. *Nature* 570: 182–8.

Snoeck, C., Lee‐Thorp, J.A., Schulting, R., De Jong, J., Debouge, W. & Mattielli, N. 2015. Calcined bone provides a reliable substrate for strontium isotope ratios as shown by an enrichment experiment. *Rapid Communications in Mass Spectrometry* 29 (1): 107–14.

Sponheimer, M. & Lee-Thorp, J.A. 1999. Oxygen isotopes in enamel carbonate and their ecological significance. *Journal of Archaeological Science* 26 (6): 723–8.

Tafuri, M.A., Craig, O.E. & Canci, A. 2009. Stable isotope evidence for the consumption of millet and other plants in Bronze Age Italy. *American Journal of Physical Anthropology* 139 (2): 146–53.

Van-Klinken, G. 1999. Bone quality indicators for paleodietary and radiocarbon measurements. *Journal of Archaeological Science* 26: 687–95.

Walker, P.L. 2005. Greater sciatic notch morphology: sex, age, and population differences. *American Journal of Physical Anthropology* 127 (4): 385‒91.

Walker, P.L. 2008. Sexing skulls using discriminant function analysis of visually assessed traits. *American Journal of Physical Anthropology* 136 (1): 39‒50.

Walsh, S. *et al*. 2013. The HIrisPlex system for simultaneous prediction of hair and eye colour from DNA. *Forensic Science International: Genetics* 7 (1): 98–115.

Weissensteiner, H. *et al*. 2016. HaploGrep 2: mitochondrial haplogroup classification in the era of high-throughput sequencing. *Nucleic Acids Research* 44: 58–63.

**Supplementary figure captions**

Figure S1: PCA with Iron Age samples available in Europe.

Figure S2: Percentage of Neolithic, WHG and Steppe Ancestry in samples from Dolge Njive.

Figure S3: Top: Pairwise allelic mismatch rate values across sliding windows of 10 Mb on the X-chromosome, moving by 1 Mb each step. The red dotted arrow indicates a shared DNA segment between I5687 and the three brothers (I22935, I5686 and I5685). Bottom: A model of DNA sharing on the X-chromosome between the four siblings (I5684, I22935, I5686 and I5685) and their parents that fits the mismatch rate patterns. We also include I5687 and his mother under the scenario in which I5687 is a maternal cousin of the four siblings. Different colours represent DNA segments inherited from different ancestors.

**Supplementary table captions**

Table S1: Genetic data by library information.

Table S2: Genetic data by sample information.

Table S3: Isotope data for Barrow 1 at Dolge njive.

Table S4: Age categories used (following Buikstra and Ubelaker 1994).

Table S5: Burial numbers with context and small find numbers.

**SUPPLEMENTARY TABLES**

| Library ID | Genetic individual ID | Raw shotgun sequences | Percent mapping to human genome | Raw sequences for capture experiment | mtDNA average coverage | Sequences aligning to mtDNA post-duplication-removal | Sequences passing filters and covering targeted nuclear SNPs before duplication removal | Sequences passing filters and covering targeted nuclear SNPs after duplication removal | Average coverage on targeted autosomal SNPs | Percentage of cytosine-to-uracil deamination in final nucleotide |
| --- | --- | --- | --- | --- | --- | --- | --- | --- | --- | --- |
| S22933.Y1.E1.L1 | I22933 | 301664 | 1.8% | 35401024 | 52 | 16329 | 12338281 | 377361 | 0.31 | 12.3% |
| S22934.Y1.E1.L1 | I22934 | 339023 | 1.8% | 36650351 | 80 | 25109 | 13439229 | 146531 | 0.12 | 10.8% |
| S22935.Y1.E1.L1 | I22935 | 344758 | 2.7% | 33741712 | 180 | 48730 | 13683391 | 1263341 | 1.01 | 8.5% |
| S22936.Y1.E1.L1 | I22936 | 326898 | 0.054% | 7455206 | 30 | 8444 | 1178292 | 47739 | 0.039 | 14.0% |
| S23971.Y1.E1.L1 | I23971 | 164242 | 12% | 27142873 | 54 | 16831 | 9848872 | 1167775 | 0.98 | 12.6% |
| S5684.E1.L1 | I5684 | .. | .. | .. | .. | .. | .. | .. | .. | .. |
| S5684.E2.L1 | I5684 | 172131 | 53% | 784460 | 134 | 42606 | 5988289 | 3300388 | .. | 9.4% |
| S5684.E2.L2 | I5684 | 180287 | 53% | 181086 | 38 | 10889 | 6306172 | 4802036 | .. | 9.9% |
| S5685.E1.L1 | I5685 | .. | .. | .. | .. | .. | .. | .. | .. | .. |
| S5685.E2.L1 | I5685 | 197163 | 58% | 822774 | 140 | 43145 | 5851388 | 4224926 | .. | 9.8% |
| S5685.E2.L2 | I5685 | 222915 | 57% | 250513 | 52 | 14306 | 5758608 | 4970279 | .. | 10.4% |
| S5687.E1.L1 | I5687 | .. | .. | .. | .. | .. | .. | .. | .. | .. |
| S5687.E2.L1 | I5687 | 229376 | 69% | 393479 | 101 | 28335 | 6711816 | 5152435 | .. | 10.2% |
| S5687.E2.L2 | I5687 | 213652 | 66% | 328537 | 87 | 24133 | 5749346 | 5192989 | .. | 10.4% |
| S5688.E1.L1 | I5687 | .. | .. | .. | .. | .. | .. | .. | .. | .. |

Table S1 Genetic data by library information

| Genetic ID | I22933 | I22934 | I22935 | I22936 | I23971 | I5684 | I5685 | I5686 | I5687 |
| --- | --- | --- | --- | --- | --- | --- | --- | --- | --- |
| Barrow | 3 | 1 | 1 | 2 | 1 | 1 | 1 | 1 | 1 |
| Burial | 1 | 5 | 3b | 1 | 2 | 1 | 3a | 4 | 6 |
| Skeletal element | tooth | tooth | tooth | bone | petrous | petrous | petrous | petrous | petrous |
| LibraryID(s) | S22933.Y1.E1.L1 | S22934.Y1.E1.L1 | S22935.Y1.E1.L1 | S22936.Y1.E1.L1 | S23971.Y1.E1.L1 | S5684.E1.L1, S5684.E2.L1, S5684.E2.L2 | S5685.E1.L1, S5685.E2.L1, S5685.E2.L2 | S5686.E1.L1 | S5687.E1.L1, S5688.E1.L1, S5687.E2.L1, S5687.E2.L2 |
| Average coverage on targeted autosomal SNPs | 0.309 | 0.117 | 1.006 | 0.039 | 0.985 | 10.206 | 13.245 | 6.419 | 19.544 |
| Number of autosomal SNPs covered at least once | 300267 | 127732 | 640596 | 44661 | 559866 | 1011266 | 1002893 | 871455 | 1006818 |
| Genetic sex | Male | Male | Male | Male | Female | Female | Male | Male | Male |
| Y haplogroup in ISOGG v15.73 notation | R1b1a1b1a1a | R1b1a1b | R1b1a1b1a1a2b1 | R1b1a1b | n/a (female) | n/a (female) | R1b1a1b1a1a2b1c2 | R1b1a1b1a1a2b1 | R1b1a1b1a1a2b1c2 |
| mtDNA haplogroup from haplogrep | H5a6 | H | H1e5a | H1ba | H1e5a | H1e5a | H1e5a | H1e5a | H1e5a |
| mtDNA match rate to consensus sequence from contamix | [0.972,0.990] | [0.986,0.999] | [0.993,1.000] | [0.980,0.998] | [0.977,0.994] | [0.98,0.996] | [0.981,0.997] | [0.969,0.991] | [0.99,1] |
| Damage rate in first nucleotide on sequences overlapping targets | 0.123 | 0.108 | 0.085 | 0.14 | 0.126 | .. | .. | 0.086 | .. |
| Ratio of Y to Y+X sequences | 0.395 | 0.39 | 0.41 | 0.399 | 0.014 | .. | .. | .. | .. |
| X-chromosome contamination from ANGSD (95% CI truncated at 0) | [0.000,0.024] | n/a (too little data) | [0.002,0.011] | n/a (too little data) | n/a (female) | n/a (female) | [0.002,0.007] | [0.001,0.007] | [0.002,0.007] |

Table S2 Genetic data by sample information

| **Burial** | **Element** | **δ^13^C _COL_**  **_VPDB_ ‰** | **δ^15^N _AIR_ ‰** | **C: N** | **δ^13^C_CARB_ _VPDB_ ‰** | **∆ δ^13^C _CARB -COL_** | **δ^18^O_CARB VSMOW_ ‰** | **Sr conc.**  **ppm** | **^87^Sr/^86^Sr norm** |
| --- | --- | --- | --- | --- | --- | --- | --- | --- | --- |
| 1 | tooth | -14.8 | 8.9 | 3.2 | -9.2 | 5.6 | 22.3 | 135 | 0.7095 |
| 1 | long bone | -15.7 | 8.0 | 3.1 |  |  |  |  |  |
| 2 | tooth | -15.6 | 8.8 | 3.3 | -7.9 | 7.7 | 22.0 | 165 | 0.7097 |
| 2 | rib | -16.5 | 8.3 | 3.3 |  |  |  |  |  |
| 2 | long bone | -16.6 | 8.3 | 3.2 |  |  |  |  |  |
| 3a | tooth | -15.8 | 8.9 | 3.2 | -9.8 | 6.0 | 22.8 | 59 | 0.7100 |
| 3a | rib | -15.5 | 8.1 | 3.3 |  |  |  |  |  |
| 3a | long bone | -15.1 | 8.5 | 3.1 |  |  |  |  |  |
| 3b | tooth | -14.9 | 9.5 | 3.2 | -8.5 | 6.4 | 21.8 | 121 | 0.7100 |
| 3b | rib | -15.0 | 8.6 | 3.2 |  |  |  |  |  |
| 4 | tooth | -13.6 | 9.5 | 3.2 | -7.3 | 6.2 | 22.2 | 86 | 0.7101 |
| 4 | rib | -16.3 | 9.0 | 3.5 |  |  |  |  |  |
| 4 | long bone | -14.7 | 9.1 | 3.2 |  |  |  |  |  |
| 5 | tooth | -15.9 | 8.7 | 3.2 | -7.2 | 8.7 | 21.1 | 226 | 0.7092 |
| 5 | rib | -15.3 | 7.9 | 3.2 |  |  |  |  |  |
| 5 | long bone | -15.3 | 8.3 | 3.2 |  |  |  |  |  |
| 6 | rib | -15.4 | 8.3 | 3.2 |  |  |  |  |  |

Table S3 Isotope data for Barrow 1 at Dolge njive

| **Age category** | **Age range** |
| --- | --- |
| Young adult | c.20-35 years |
| Middle adult | c.36-50 years |
| Mature adult | c.50+ years |
| Adult | c.18+ years |

Table S4 Age categories (following Buikstra and Ubelaker 1994).

| **Burial** | **Context** | **Small find no.** |
| --- | --- | --- |
| **Barrow 1** | | |
| 1 | 1763 | 1708 (1775) |
| 2 | 1746 | 1781 |
| 3a | 1748 | 2603 |
| 3b | 1748 | 2604 |
| 4 | 2644 | 2680a |
| 5 | 2650 | 2900 |
| 6 | 2653 | 2903 |
| **Barrow 2** | | |
| 1 | n/a | 2361/6 |
| **Barrow 3** | | |
| 1 | n/a | 1883 |

Table S5 Burial numbers with context and small find numbers
