# Supplementary figures and images for: Kinship practices in Early Iron Age southeast Europe: genetic and isotopic analysis of burials from the Dolge njive barrow cemetery, Dolenjska, Slovenia
Source: Antiquity. Author manuscript; Available in PMC 2024 Nov 24. (PMC11586097; doi:10.15184/aqy.2023.2)

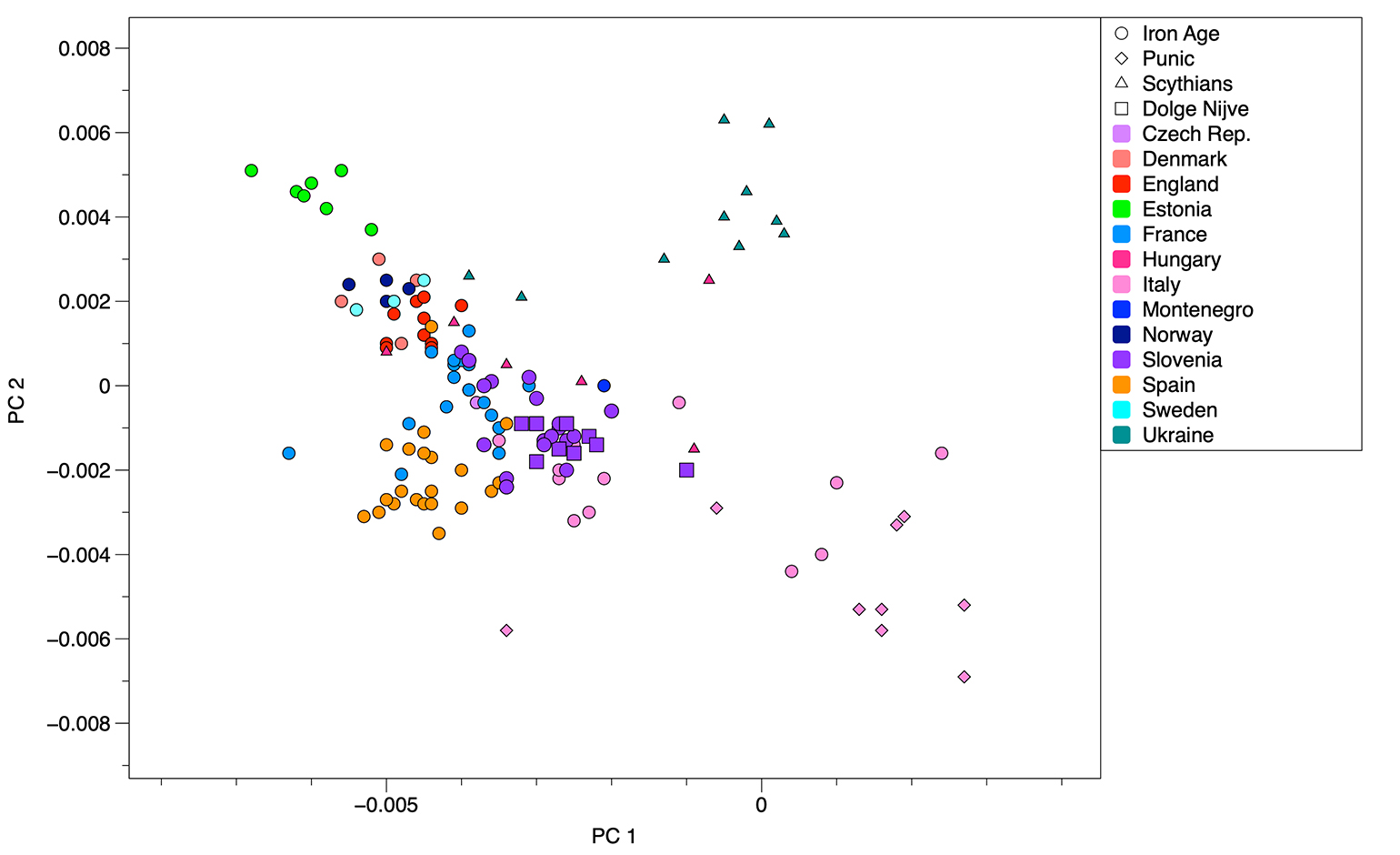

Supplement: Figure S2 [file NIHMS2033570-supplement-Figure_S2.jpg]

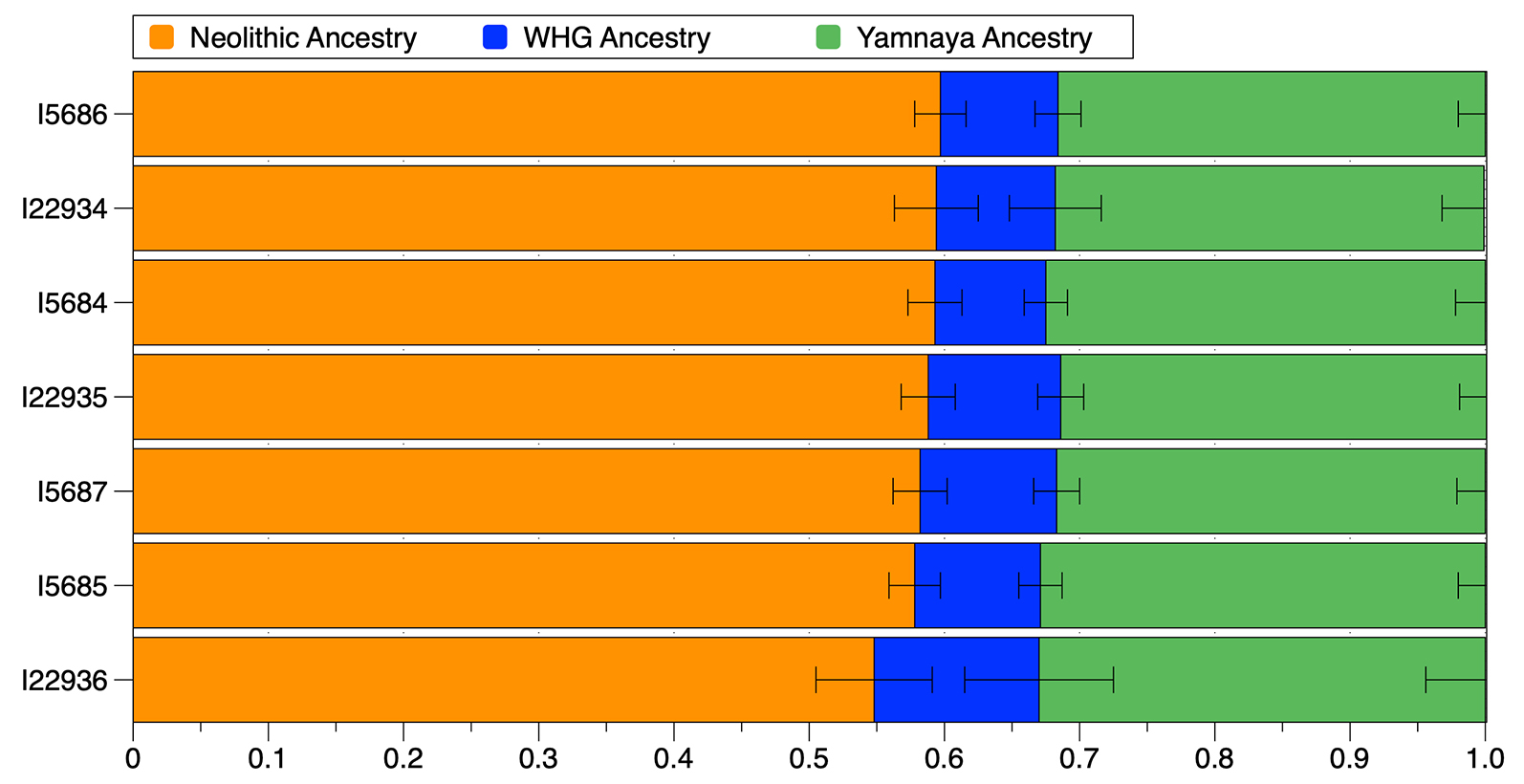

Supplement: Figure S3 [file NIHMS2033570-supplement-Figure_S3.jpg]

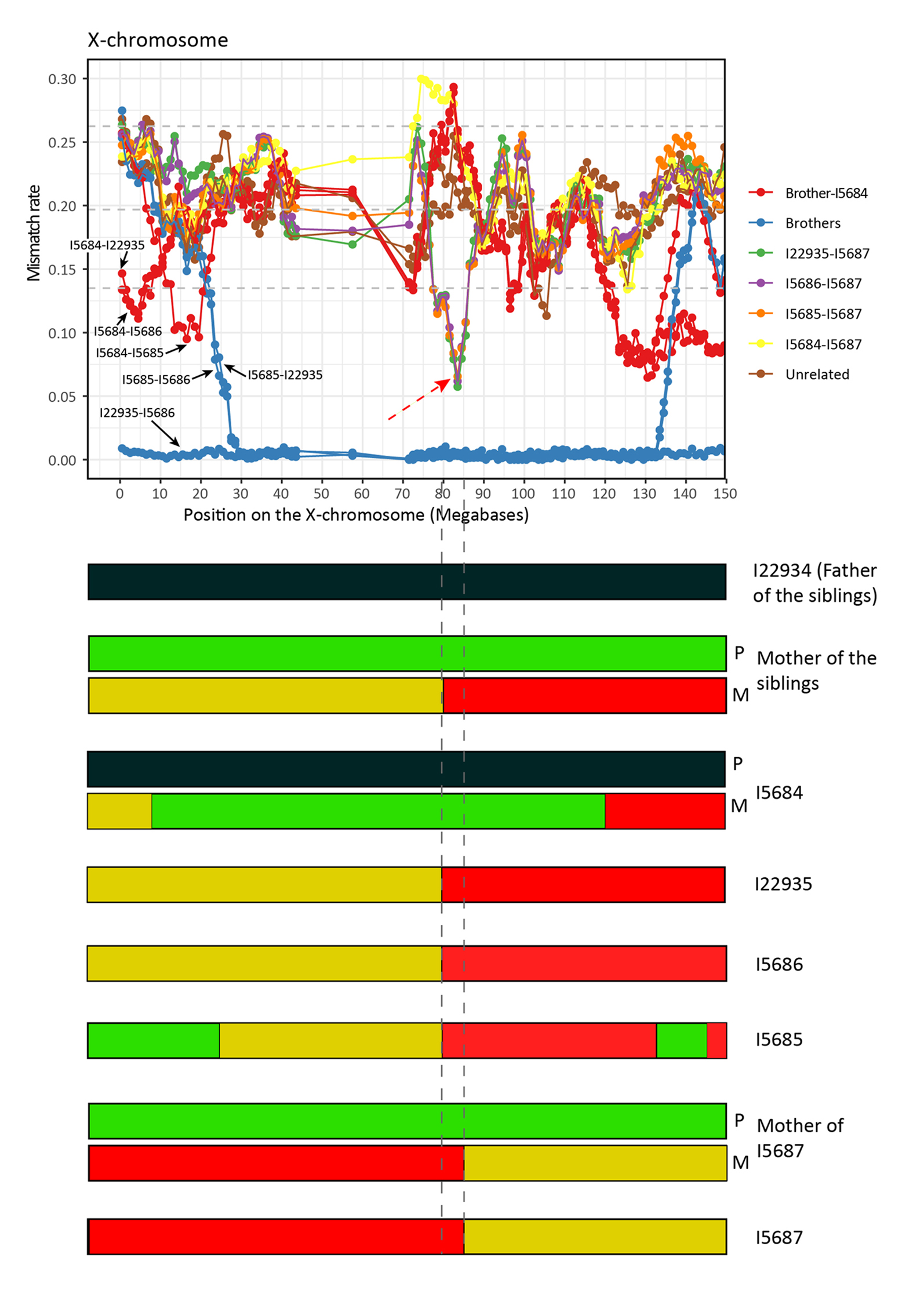

Supplement: Figure S1 [file NIHMS2033570-supplement-Figure_S1.jpg]
